# Semi-Supervised Segmentation Framework for Gastrointestinal Lesion Diagnosis in Endoscopic Images

**DOI:** 10.3390/jpm13010118

**Published:** 2023-01-05

**Authors:** Zenebe Markos Lonseko, Wenju Du, Prince Ebenezer Adjei, Chengsi Luo, Dingcan Hu, Tao Gan, Linlin Zhu, Nini Rao

**Affiliations:** 1 Center for Informational Biology, University of Electronic Science and Technology of China, Chengdu 610054, China; 2 School of Life Science and Technology, University of Electronic Science and Technology of China, Chengdu 610054, China; 3 School of Public Health, College of Health Sciences and Medicine, Dilla University, Dilla P.O. Box 419, Ethiopia; 4 Department of Computer Engineering, Kwame Nkrumah University of Science and Technology, Kumasi AK-039-5028, Ghana; 5 Digestive Endoscopic Center of West China Hospital, Sichuan University, Chengdu 610017, China

**Keywords:** gastrointestinal disease diagnosis, gastrointestinal lesion segmentation, endoscopic images, semi-supervised learning, computer-aided diagnosis, generative adversarial learning, deep learning

## Abstract

Background: Accurate gastrointestinal (GI) lesion segmentation is crucial in diagnosing digestive tract diseases. An automatic lesion segmentation in endoscopic images is vital to relieving physicians’ burden and improving the survival rate of patients. However, pixel-wise annotations are highly intensive, especially in clinical settings, while numerous unlabeled image datasets could be available, although the significant results of deep learning approaches in several tasks heavily depend on large labeled datasets. Limited labeled data also hinder trained models’ generalizability under fully supervised learning for computer-aided diagnosis (CAD) systems. Methods: This work proposes a generative adversarial learning-based semi-supervised segmentation framework for GI lesion diagnosis in endoscopic images to tackle the challenge of limited annotations. The proposed approach leverages limited annotated and large unlabeled datasets in the training networks. We extensively tested the proposed method on 4880 endoscopic images. Results: Compared with current related works, the proposed method validates better results (Dice similarity coefficient = 89.42 ± 3.92, Intersection over union = 80.04 ± 5.75, Precision = 91.72 ± 4.05, Recall = 90.11 ± 5.64, and Hausdorff distance = 23.28 ± 14.36) on the challenging multi-sited datasets, confirming the effectiveness of the proposed framework. Conclusion: We explore a semi-supervised lesion segmentation method to employ the full use of multiple unlabeled endoscopic images to improve lesion segmentation accuracy. Experimental results confirmed the potential of our method and outperformed promising results compared with the current related works. The proposed CAD system can minimize diagnostic errors.

## 1. Introduction

Gastrointestinal (GI) tract cancers affect the human digestive system, resulting in one of the most critical healthcare problems [1,2]. According to report [3,4], esophageal cancer, colorectal cancer, and stomach cancer are the three most common GI cancers with the highest incidence and mortality rates [5,6]. Endoscopy is the primary method for the examination of the GI tract; gastroscopy examines the upper GI tract, whereas colonoscopy examines the bowel and rectum [4,7]. Endoscopic examinations typically require expensive, highly standardized equipment and expertise.

Nowadays, automatic segmentation of lesions and anatomical structures in biomedical imaging has led to improvements in accurately diagnosing medical conditions. Advances in deep learning (DL), particularly convolutional neural networks (CNNs), have resulted in significant progress in several vision-related tasks [8,9,10], frequently attaining human-level competitiveness in recognizing lesions in tissues and delineating heart structures [11], and segmenting colorectal polyps [10] and esophageal cancer [7] in GI diseases [12,13]. The success of DL usually depends on the availability of massive labeled high-quality datasets [11]. Accordingly, several deep supervised segmentation models are implemented in fully supervised approaches such as U-Net [14], FCN [15], and UNet++ [16]. However, it is difficult and costly to obtain annotated data in the biomedical imaging environment [17]. Furthermore, even where data are available, there is the need for domain experts to manually produce annotations: a process that is often tedious and impractical on large scale.

One way of addressing this limitation is to use a semi-supervised learning (SSL) approach. SSL is a hybrid of supervised and unsupervised learning methods where a model is trained on a large dataset where only a small volume of the data is annotated. Typically, these data will be the targets linked with some samples [18]. Thus, by reducing the need for annotating large volumes of medical images, an efficient SSL framework offers an attractive alternative to supervised DL approaches in automatic segmentation applications in biomedical imaging. To effectively validate the quality of a SSL method, a few studies [19,20,21] have applied a generative adversarial network (GAN) [18]. GANs comprise a generator network and a discriminator network [18,20].

The lack of sufficiently labeled images for classical DL techniques means that there is the need for SSL techniques that (1) require little supervision [20] and (2) can self-evaluate in an unsupervised manner [22,23]. In this work, we propose a robust SSL framework for training deep models with small labeled training samples. Furthermore, we utilize adversarial generative modeling similar to [23] for GI lesion segmentation tasks [21].

Inspired by [20], this work introduces an adversarial-based semi-supervised learning approach into a densely connected CNN for GI automatic lesion segmentation. The key contributions of this work are summarized as follows:

(1) We propose a GAN-based semi-supervised GI lesion segmentation framework that uses reasonably small labeled endoscopic images.
(2) We demonstrate a full use of numerous unlabeled GI datasets to improve lesion segmentation accuracy.
(3) The proposed framework was tested on five multi-sited datasets from different centers and integrated the predicted result to improve the segmentation performance through generative adversarial training.
(4) The proposed method outperforms baseline supervised segmentation models as well as other related semi-supervised segmentation frameworks.

The next section of this work is organized as follows. In the following section, we describe the materials and methods of our proposed GAN-based semi-supervised framework. Section 3 deals with the experimental setup. In the consecutive section, we describe the results and discussion and conclude in Section 5.

## 2. Materials and Methods

### 2.1. GI image Datasets

We collected 4880 GI images in total from a private hospital and publicly available sources to evaluate our method. The details of each dataset are as follows:

#### 2.1.1. West China Hospital Digestive Endoscopy Center Dataset

We employed 2112 GI images of 484 patients collected and verified by gastroenterologists from the Digestive Endoscopy Center of the West China Hospital in Sichuan, China. The images were stored as RGB color channel in JPEG format. Original images were captured at a resolution of 384 × 384 pixels. Lesion areas were marked and labeled as ground truth (GT) by gastroenterologists. The GT images are stored as black and white binary images and also in JPEG format. From 2112 GI images, only 192 images were GT, and eighty percent of the datasets were used for training with the remainder used for testing. Approval from the medical ethical review committee of the University of Electronic Science and Technology of China (UESTC) and West China Hospital and informed patients’ consent were obtained.

#### 2.1.2. Public Datasets

**Figure 1:**
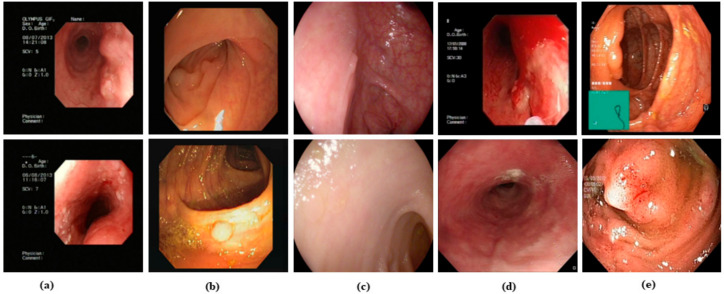
Sample raw GI images. (**a**) GI images with the small and large lesion region (lab), (**b**) CVC-ClinicDB polyp images with small and large lesion region [24], (**c**) ETIS-LaribPolypDB polyp images [25], (**d**) EDA2019 challenge images [26], and (**e**) Kvasir-SEG images dataset with small and large lesions region [27].

To ensure a robust framework, we used four related public datasets. Firstly, CVC-ClinicDB [24] data were used to train and validate the network. CVC-ClinicDB is a database that contains 612 images of 384 × 288-pixel spatial resolution. Secondly, the ETIS-LaribPolypDB [25] consisting of 196 polyp images from 34 different video sequences with labeled GT images of size of 1225 × 966 pixels was used. The third dataset was collected from the endoscopy artifact detection (EAD2019) [26] challenge for semantic segmentation with seven different classes (specularity, artifact, saturation, contrast, bubbles, blur, and instrument). The images were in different resolutions, including 1920 × 1080, 1349 × 1079, and 295 × 299 in JPG format. From 2622 images, only 960 related images with lesions were used for semi-supervised training. The fourth dataset used in this study was the Kvasir-SEG dataset [27], which has one thousand polyp images and their corresponding GT. The resolution of the images varies from 332 × 487 to 1920 × 1072 pixels which are encoded in JPEG. Samples of raw images are shown in Figure 1.

### 2.2. Methods

This study discusses an SSL method based on adversarial generative training techniques for GI lesions segmentation. Our framework uses a weighted amalgamation of losses.

The general framework of the semi-supervised GI lesion segmentation is divided into four main consecutive steps, with the output of each step being the input to the subsequent step. The main detailed steps are explained as follows.

Step 1: Preprocessing

In the first stage, unnecessary background and artifacts were removed from each original dataset denoted *đ*. Moreover, due to the varying nature of training images sizes, all training images were resized into 192 × 192 to fit the input model we used. All the resized RGB images were converted into a PNG file during Step 1.

Step 2: Supervised Training

The preprocessed dataset from Step 1, denoted *đ_1_*, was used to train the network in this step in a supervised manner. The outputs of Step 2, i.e., the model weight from supervised learning, denoted *đ_2_*, and the unlabeled datasets, denoted *đ_4,_,* were forwarded to Step 3 for semi-supervised training.

Step 3: Adversarial Training

**Figure 2:**
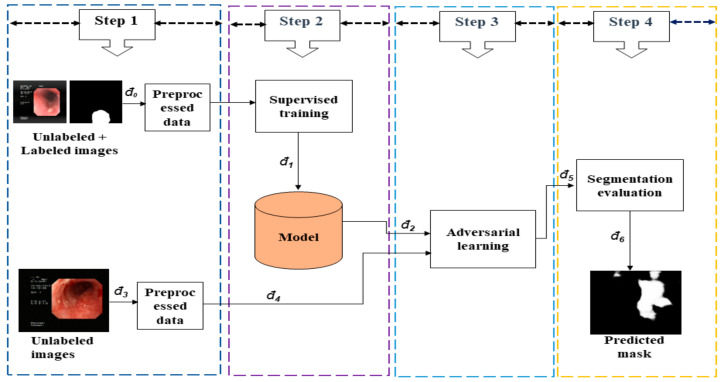
Semi-supervised GI lesion segmentation framework.

**Figure 3:**
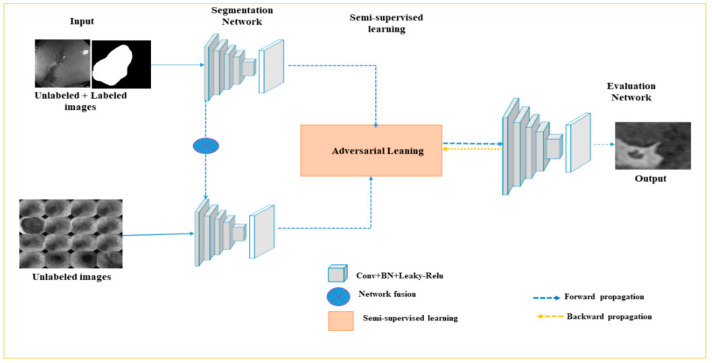
Overview of proposed semi-supervised GI lesion segmentation architecture. Initially, a segmentation network (SN) is applied by using labeled data and corresponding GT data in a fully supervised fashion. Then, an evaluation network (EN) is introduced to give different scores to the segmentation of unlabeled and unlabeled data.

As shown in Figure 2, the input to the semi-supervised module consists of the unlabeled dataset, *đ_4_*, and the pre-trained model, *đ_2_*. The segmentation probability map is generated by adversarial training in Step 3. The output, *đ _6_*, will be reserved in Step 4, for further evaluation. Subsequently, the EN evaluates the input’s segmentation quality, which comprises the GT from the labeled images, and the predicted output from labeled and unlabeled images. The proposed network can accomplish adversarial learning between the SN and EN based on the test scores. Through adversarial training, the predicted maps generated from SN can be close to the GT. Figure 3 shows our semi-supervised network architecture which comprises SN and EN. Initially, the SN is trained with labeled data in a supervised learning fashion.

Step 4: Testing Model

In Figure 3, the EN requires two inputs (i.e., GI images and the corresponding region of interest (ROI) segmentation maps). The ROI segmentation is fed with GT for testing for the labeled images. Finally, the performance results are determined by the EN.

#### Loss Function

The loss function of the network is defined as in [9]. Both the labeled GI image $X_{ı}$ and the unlabeled GI images $X_{u}$ are of size H × W. The segmentation and the evaluation networks are denoted by Seg(•) and Ev(•), respectively. GT images labeled by the physicians are symbolized as Yι. Yι is of size H × W and incorporates two channels and *Ŷ* denotes the predicted maps. The model is implemented by reducing the following loss function as follows:

(1) $$\ell_{S}=\ell_{seg}+\lambda_{\mathrm{adv}}\ell_{\mathrm{adv}}$$

where $\ell_{\mathrm{seg}}$ denotes the supervised loss and $\ell_{\mathrm{adv}}$ adversarial loss. $\lambda_{\mathrm{adv}}$ denotes the weight of the adversarial learning model. The loss function $L_{\mathrm{seg}}$ regulates whether the predicted probability generated from the input labeled image is similar to the GT, and it is formulated as:

(2) $$L_{seg}(X_{ı},Y_{u};\;\;\theta_{S})=\;L_{dce}(Seg(X_{ı}),\;Y_{u})\;\;+L_{bce}\;(Seg(X_{ı}),\;Y_{u})$$

where $\theta_{S}$, $L_{bce}$ and $\ell_{\mathrm{adv}}$ denotes the parameters of the SN, binary cross-entropy loss (b*ce*), and adversarial loss calculated to approximate the performance from either the labeled or unlabeled images. The $\ell_{\mathrm{adv}}$ is expressed as:

(3) $$\ell_{\mathrm{adv}}(X_{ı},X_{u};\theta_{S})=\;\lambda_{ı}\;.L_{bce}(\mathrm{Ev}\left(X_{ı},Seg\left(X_{ı}\right),1\right)+\lambda_{u}.\;L_{bce}(\mathrm{Ev}(X_{u},\;\left(Seg\left(X_{u}\right),1\right)$$

(4) $$\ell_{E}=(X_{ı},\;X_{u},\;Y_{ı};{\;\theta }_{E})=\;L_{bce}(\mathrm{Ev}\left(X_{ı},\;Y_{ı}\right),1+\;\lambda_{ı}\;.L_{bce}\left(\mathrm{Ev}\left(X_{ı},\;Seg\left(X_{ı}\right)\right),0\right)+\;\lambda_{u}\;.L_{bce}\left(\mathrm{Ev}\left(X_{u},\;Seg\left(X_{u}\right)\right),0\right)$$

where $\theta_{E}$ represents the parameters of the EN. $\lambda_{ı}$ and $\lambda_{u}$ correspond to the loss coefficients of the labeled and unlabeled data, respectively.

## 3. Experimental Setup

### 3.1. Implementation Details

We implemented the *BCE* loss during the supervised training with stochastic gradient descent (SGD) [28] optimizer at a learning rate (LR) of 0.001. The semi-supervised loss is to train the semi-supervised models, and SN is trained with SGD with a LR of 0.001 and 0.0001 weight decay. The networks are optimized with Adam optimizer at an initial learning rate of 0.001, batch size 16, and 4000 epochs. The network was implemented in Python 3.6.4 and Tensorflow (https://www.tensorflow.org (accessed on 10 June 2021)) [29]. Experiments were implemented on Ubuntu 16.04.6 LTS (server-based), and the system was equipped with four GPUs of NVIDIA GeForce RTX 2080Ti with 11 GB memory each.

### 3.2. Evaluation Metrics

The following five pixel-level evaluation metrics are utilized to compare the segmentation performance of the proposed method. Labeled datasets by physicians were used as GT; five different evaluation metrics were utilized to evaluate the performance, namely: Dice similarity coefficient (DSC), intersection over union (IOU), precision (Pre), recall (Rec), and Hausdorff distance (HDist). DSC is the most common metric for comparing the predicted segmentation and GT. IOU is a typical metric to measure a prediction, and it calculates the overlaps between the prediction and its corresponding GT [30]. HDist measures how far the prediction and the GTs are from each other. The lower the HDist is, the better the image difference, and a closer HDist to zero indicates a better image [9]. DSC, IOU, Pre, and Rec were used to validate the overlap between the GT and prediction. HDist was used to evaluate the distance between a model prediction (P) lesion boundary and the GT. The performance indicators implemented are shown below:

(5) $$Pre=\frac{TP}{TP+FP}$$

(6) $$Pre=\frac{TP}{TP+FP}$$

(7) $$DSC\;\left(A,B\right)=\frac{2\times \left|A\cap B\right|}{\left|A\right|+\left|B\right|}=\frac{2\times TP}{2\times TP+FP+FN}$$

(8) $$IOU\;\left(A,B\right)=\frac{A\cap B}{A\cup B}=\frac{TP}{TP+FP+FN}$$

where *TP*, *FP*, and *FN* represent the number of true positive pixels, false-positive pixels, and false-negative pixels in segmentation regions. A and B represent the ratio between the overlapped area A∩B over the total area A∪B corresponded by the two boxes.

(9) $$HDist\;\left(A,B\right)\;\underset{a\in A}{\max}\;\{\underset{b\in B}{\min}\left\{\;d\left(a,b\right)\right\}\}$$

where *a* and *b* are points of *A* (GT contour) and *B* (predicted contour), respectively; *d*(*a*, *b*) is a metric between these points. In this case, we take *d*(*a*, *b*) as the Euclidian distance between *a* and *b*.

## 4. Results and Discussion

In this part, we describe performance comparisons for baseline network trained in a supervised fashion and then the semi-supervised technique for adversarial training. Fixed images with GT were utilized for all unlabeled images and trained adversarially for lesion segmentation.

### 4.1. Comparisons Using the Limited Labeled GI Datasets

Table 1 shows a comparison of our model performance on four GI datasets. To confirm the proposed approach’s effectiveness, we employed a limited GT for each dataset in a supervised learning fashion. Finally, the proposed method had promising results in all datasets’ differences. Different GI datasets prepared by different physicians were confirmed for the next level. Kvasir-SEG dataset, Ref [27] in terms of the mean value and SD (DSC = 84.65 ± 18.09, IOU = 75.14 ± 18.39, Pre = 86.0 ± 18.81, Rec = 85.0 ± 19.30, and HDist = 35.14 ± 20.63) respectively, achieved better results than other datasets. This was a probability of the total number of images used during training. A higher recall (Rec = 91.20 ± 8.45) result was performed on the ETIS dataset [25]. The average value of HDist (HDist = 30.02 ± 19.11) on our dataset showed fewer distance results than other datasets.

### 4.2. Supervised Learning Comparisons

After preprocessing, we compared the baseline framework with two state-of-art works: U-Net [14] and UNet++ [16]. Each experiment was run with fixed 192 (from our dataset) labeled GI images. Input images were resized in 192 × 192 and the same evaluation metrics were utilized for all models. Three models demonstrated competitive results, as shown in Table 2. The baseline achieved a better mean value on three metrics (DSC = 82.15 ± 10.22, Pre = 84.22 ± 15.94, and HDist = 32.09 ± 26.44). The pre-trained results were utilized for further semi-supervised learning.

### 4.3. Semi-Supervised Learning Comparisons

**Figure 4:**
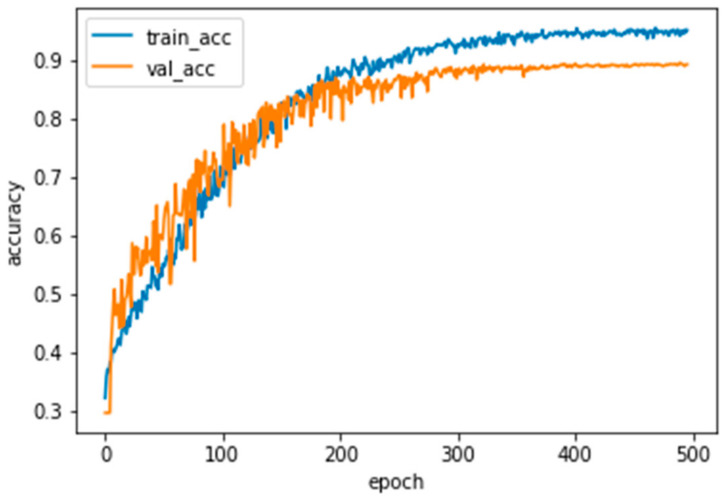
The learning curve shows accuracy performance over several epochs of the proposed model. At the first epoch, training and validation accuracy was low, with higher losses. After epoch 300, the model was slightly stable, and after epoch 400, it became stable with significant accuracy improvement.

We compared our method with two recent related deep adversarial learning-based methods on our dataset to validate the segmentation accuracy. The two comparison methods were DAN [22] and GAN [20]. To ensure the best segmentation results of each method and ensure comparativeness, the input size of the data was set as 192 × 192. The dataset was split into 192, 384, 768, 960, and 1920, respectively. Due to the unavailability of 1920 images from the EAD2019 dataset [26], only the lab dataset was employed for the final training. Using a fixed labeled dataset with GT for all, the rest of the unlabeled dataset was randomly selected for the proposed model. The learning curve of training and validation performance of the proposed method is presented in Figure 4. The proposed model achieves better results (DSC = 89.42 ± 3.92, IOU = 80.04 ± 5.75, Pre = 91.72 ± 4.05, Rec = 90.11 ± 5.64, and HDist = 23.28 ± 14.36). Almost all models achieved lower performance results on the EAD2019 dataset [26].

**Figure 5:**
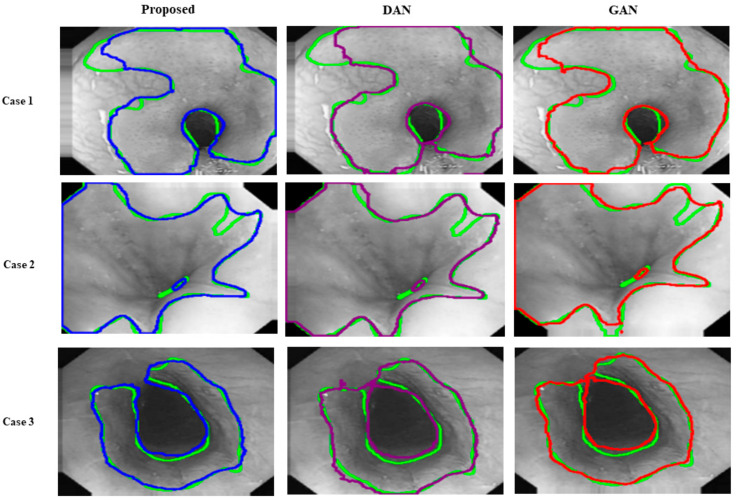
Visual samples of the segmentation performance of semi-supervised learning (**right**), (**middle**), and our method (**left**) on the test dataset. The green, yellow, purple, and red contours denote the GT, proposed, GAN, and DAN segmentation results.

Moreover, our model can achieve better GI lesion accuracy on all testing datasets. Table 3 indicates the effectiveness of our method compared with other related works. As shown in Figure 5, qualitative results also confirmed the competitive effects of the models. Accurate segmentation of ROI from GI images is essential for CAD procedures. However, due to the variability of GI lesions, it is challenging to advance accuracy with limited annotated data. The proposed GAN–based model can leverage unlabeled data to obtain better accuracy than related models [18,22]. This indicates the proposed method is applicable for lesion segmentation.

Extensive experimental results confirm that the proposed method can produce promising segmentation maps. Usually, GI images’ appearance differs across generating equipment, with an effect on GI lesion [31]. However, the proposed GAN-based approach achieves more promising results than the state-of-the-art methods, indicating the proposed method’s effectiveness on four datasets. Furthermore, the proposed model can aid clinical annotation tasks by reducing medical errors. Thus, accurate segmentation can help physicians and improve the robustness of GI lesion’s automatic diagnosis.

### 4.4. Comparison of Baseline and Proposed Models

We have performed several experiments with initial validation using two different methods. The first approach is based on a supervised way that includes U-Net [14] convolutional biomedical image segmentation and UNet++ [16], as shown in Table 2. The second method is based on a semi-supervised method utilizing the generator and discriminator network. We compared the proposed method’s performance with DAN [22] and GAN [20] in the second primary method.

## 5. Conclusions

This paper proposed an adversarial learning-based semi-supervised segmentation framework for GI lesion diagnosis in endoscopic images. Automatic segmentation of lesions from endoscopic images is crucial to assist physicians in GI digestive tract disease diagnosis. We conducted intensive experiments using five different datasets and test data evaluated using five evaluation metrics. We explore a semi-supervised lesion segmentation method to employ the full use of multiple unlabeled images to improve lesion segmentation accuracy. Extensive experimental results demonstrated the potential of our method and outperformed other related works. A proposed CAD system can assist physicians and minimize diagnostic medical errors. Improving the approaches with more robustness and generalizability to other related semi-supervised learning tasks will be the basis of future work.

## Figures and Tables

**Table 1:** Lesion segmentation comparison using the limited labeled GI datasets.

Data Source	Total	DSC (%) ± SD	IoU (%) ± SD	Pre (%) ± SD	Rec (%) ± SD	HDist (mm) ± SD
Lab	192	82.70 ± 7.71	72.10 ± 10.20	87.00 ± 9.40	81.01 ± 11.81	30.02 ± 19.11
ETIS-LaribPolypDB [25]	196	77.46 ± 17.30	67.01 ± 19.72	71.41 ± 22.02	91.20 ± 8.45	34.92 ± 31.72
CVC-ClinicDB [24]	612	84.23 ± 14.03	74.56 ± 16.12	84.01 ± 15.46	87.04 ± 13.56	32.48 ± 26.23
Kvasir-SEG [27]	1000	84.65 ± 18.09	75.14 ± 18.39	86.0 ± 18.81	85.0 ± 19.30	35.14 ± 20.63

**Table 2:** Supervised segmentation performance comparisons.

Model	DSC (%) ± SD	IOU (%) ± SD	Pre (%) ± SD	Rec (%) ± SD	HDist (mm) ± SD
U-Net [14]	81.04 ± 14.28	68.04 ± 16.01	85.15 ± 16.55	86.62 ± 15.03	36.17 ± 28.18
UNet++ [16]	81.68 ± 13.65	70.56 ± 14.32	86.22 ± 15.61	85.14 ± 15.27	33.49 ± 27.24
Baseline	82.15 ± 10.22	70.28 ± 12.04	84.22 ± 15.94	83.78 ± 14.69	32.09 ± 26.44

**Table 3:** Semi-supervised model performance results compared to GI dataset: Lab (A) data and EAD2019 challenge data (B).

Labeled/ Unlabeled Data	Model	DSC (%) ± SD		IOU (%) ± SD		Pre (%) ± SD		Rec (%) ± SD		HDis (mm) ± SD	
		A	B	A	B	A	B	A	B	A	B
192/192	DAN [22]	74.56 ± 20	69.01 ± 15.48	62.45 ± 13.08	61.30 ± 19.42	83.45 ± 13.23	71.12 ± 16.48	72.78 ± 14.05	72.96 ± 18.11	36.11 ± 23.26	39.24 ± 34.11
	GAN [20]	79.62 ± 12.56	71.23 ± 14.34	68.55 ± 12.71	63.02 ± 18.57	89.56 ± 12.32	72.34 ± 18.01	74.12 ± 14.56	73.29 ± 19.23	37.25 ± 22.34	40.11 ± 33.56
	Ours	83.10 ± 8.45	74.20 ± 13.02	71.02 ± 9.58	66.04 ± 17.21	88.24 ± 10.26	77.02 ± 16.78	81.42 ± 12.04	76.26 ± 18.63	32.33 ± 20.26	37.79 ± 31.26
192/384	DAN [22]	81.22 ± 9.08	75.48 ± 13.02	68.45 ± 11.8	65.89 ± 18.11	79.14 ± 17.56	75.64 ± 17.6	87.58 ± 13.42	79.43 ± 18.25	33.89 ± 21.13	38.26 ± 33.24
	GAN [20]	82.25 ± 8.11	76.89 ± 11.36	70.58 ± 11.15	67.1 ± 16.38	79.40 ± 16.24	76.7 ± 16.82	86.51 ± 11.42	80.02 ± 16.11	33.8 ± 20.52	36.24 ± 32.45
	Ours	83.45 ± 7.23	79.62 ± 11.01	72.81 ± 9.56	68.45 ± 16.24	86.87 ± 9.6	80.50 ± 16.32	82.47 ± 10.9	81.4 ± 15.56	28.9 ± 18.12	35.54 ± 29.33
192/768	DAN [22]	85.86 ± 8.14	80.56 ± 10.2	75.5 ± 11.37	73.01 ± 13.15	87.25 ± 7.48	80.2 ± 15.01	79.9 ± 12.06	80.56 ± 15.1	29.4 ± 19.15	33.6 ± 26.37
	GAN [20]	85.39 ± 7.4	80.25 ± 10.16	75.58 ± 10.39	73.89 ± 13.7	89.61 ± 6.51	82.62 ± 14.88	80.12 ± 11.45	81.63 ± 13.5	30.8 ± 18.9	33.06 ± 28.4
	Ours	86.65 ± 5.9	82.8 ± 9.4	76.72 ± 8.7	75.8 ± 10.69	92.74 ± 5.89	84.56 ± 11.4	81.85 ± 9.7	86.64 ± 12.7	25.4 ± 16.72	31.37 ± 22.46
192/960	DAN [22]	86.8 ± 7.24	82.34 ± 9.5	77.8 ± 10.36	74.6 ± 12.65	83.5 ± 7.62	83.8 ± 12.96	91.02 ± 11.3	85.45 ± 12.39	25.6 ± 18.4	31.2 ± 27.5
	GAN [20]	86.7 ± 7.31	82.81 ± 9.76	76.9 ± 9.7	76.52 ± 13.2	92.03 ± 7.46	84.72 ± 11.34	82.36 ± 10.4	85.41 ± 11.7	24.7 ± 17.42	30.61 ± 22.63
	Ours	88.6 ± 4.08	83.81 ± 8.4	79.72 ± 6.42	77.36 ± 9.48	91.69 ± 4.78	87.24 ± 10.85	86.09 ± 5.91	89.28 ± 11.42	24.93 ± 15.83	30.45 ± 21.05
192/1920	DAN [22]	87.03 ± 6.15	-	78.95 ± 9.36	-	85.62 ± 6.78	-	89.63 ± 10.13	-	24.68 ± 16.59	-
	GAN [20]	87.49 ± 5.63	-	78.41 ± 8.74	-	92.81 ± 6.29	-	85.46 ± 9.2	-	24.05 ± 16.17	-
	Ours	89.42 ± 3.92	-	80.04 ± 5.75	-	91.72 ± 4.05	-	90.11 ± 5.64	-	23.28 ± 14.36	-

## Data Availability

In this study, we used two primary data sources (hospital and public), available in references. Except for the hospital dataset, which will be available upon request made to a corresponding author due to data privacy restrictions, the rest of the public datasets are available at https://datasets.simula.no/kvasir/ (accessed on 10 March 2022); https://polyp.grand-challenge.org/CVCClinicDB (accessed on 16 February 2021); https://doi.org/10.1007/s11548-013-0926-3 (accessed on 17 February 2021); https://doi.org/10.17632/C7FJBXCGJ9.1 (accessed on 2 April 2022), and https://ead2019.grand-challenge.org (accessed on 2 April 2022).

## Abbreviations

CAD	computer-aided diagnosis
CNN	convolutional neural network
DL	deep learning
DSC	dice similarity coefficient
EN	evaluation network
FCN	fully connected network
GI	gastrointestinal
GT	ground truth
HDis	Hausdorff distance
GAN	generative adversarial network
LR	learning rate
Pre	precision
Rec	recall
ROI	region of interests
SN	segmentation network
SSL	semi-supervised learning
SD	standard deviation
SGD	stochastic gradient descent
